# A Multi-Year Study of Mycotoxin Co-Occurrence in Wheat and Corn Grown in Ontario, Canada

**DOI:** 10.3390/toxins16080372

**Published:** 2024-08-22

**Authors:** Megan J. Kelman, J. David Miller, Justin B. Renaud, Daria Baskova, Mark W. Sumarah

**Affiliations:** 1 London Research and Development Centre, Agriculture and Agri-Food Canada, 1391 Sandford Street, London, ON N5V 4T3, Canada; megan.kelman@agr.gc.ca (M.J.K.); justin.renaud@agr.gc.ca (J.B.R.); daria.baskova@agr.gc.ca (D.B.); 2 Department of Chemistry, Carleton University, 1125 Colonel By Drive, Ottawa, ON K1S 5B6, Canada; david.miller@carleton.ca; 3 Department of Chemistry, University of Western Ontario, 1151 Richmond Street, London, ON N6A 5B7, Canada

**Keywords:** mycotoxins, deoxynivalenol, trichothecenes, sterigmatocystin, LC-MS, diacetoxyscirpenol

## Abstract

Mycotoxin emergence and co-occurrence trends in Canadian grains are dynamic and evolving in response to changing weather patterns within each growing season. The mycotoxins deoxynivalenol and zearalenone are the dominant mycotoxins detected in grains grown in Eastern Canada. Two potential emerging mycotoxins of concern are sterigmatocystin, produced by *Aspergillus versicolor*, and diacetoxyscirpenol, a type A trichothecene produced by a number of *Fusarium* species. In response to a call from the 83rd Joint Expert Committee on Food Additives and Contaminants, we conducted a comprehensive survey of samples from cereal production areas in Ontario, Canada. Some 159 wheat and 160 corn samples were collected from farms over a three-year period. Samples were extracted and analyzed by LC-MS/MS for 33 mycotoxins and secondary metabolites. Ergosterol was analyzed as an estimate of the overall fungal biomass in the samples. In wheat, the ratio of DON to its glucoside, deoxynivalenol-3-glucoside (DON-3G), exhibited high variability, likely attributable to differences among cultivars. In corn, the ratio was more consistent across the samples. Sterigmatocystin was detected in some wheat that had higher concentrations of ergosterol. Diacetoxyscirpenol was not detected in either corn or wheat over the three years, demonstrating a low risk to Ontario grain. Overall, there was some change to the mycotoxin profiles over the three years for wheat and corn. Ongoing surveys are required to reassess trends and ensure the safety of the food value chain, especially for emerging mycotoxins.

## 1. Introduction

The mycotoxin contamination of food and feed is a major economic challenge for the small grains value chain in Canada. The risks associated with mycotoxins in grain were first appreciated following the recognition of ergot toxicosis in the 17th century, then again following the discovery of citrinin in 1931, the isolation of deoxynivalenol (DON: also known as vomitoxin) in 1972, and the discovery of fumonisin in 1988 [1,2]. In Canada, early reports of animal toxicoses from grains with Fusarium head blight (FHB) appeared in the literature in the late 19th century [3]. The causative fungal agent of FHB in wheat and other small grains, *Fusarium graminearum*, is also responsible for Gibberella ear rot in corn [4]. As chemical standards became available, reports of mycotoxins in feed and food, particularly ochratoxin (OTA) and T-2/HT-2 toxins, started to appear in the early 1970s [5,6,7,8,9]. The catastrophic FHB of 1980 in Southern Ontario produced a crop that was highly contaminated by DON [10]. This resulted in increased efforts to monitor and understand the toxicity of mycotoxins of importance to Canada. Wheat is susceptible to head blight if it is warm and wet at anthesis, while corn is prone to ear rot if those conditions occur around silk emergence [11].

Since the late 1970s, the pattern of the mycotoxin contamination in Ontario changed. In the 1970s, the *Fusarium graminearum* toxin zearalenone (ZEN) was common in corn and to a lesser extent in small grains [12,13]. Zearalenone is biosynthesized under cooler conditions, whereas the same fungus produces deoxynivalenol (DON) under warmer—but not hot—conditions [14,15]. As temperatures in Ontario increased from the 1980s to the present, DON came to dominate in crops during years where Fusarium head blight or Gibberella ear rot occurred [15,16,17]. Aside from DON, the precursor 15-acetyldexoynivalenol (15-ADON) as well as the plant-produced glycoside deoxynivalenol-3-glucoside (DON-3G) are common in infected wheat and corn in Ontario. The acetylated DON derivative, 3-ADON, has come to dominate parts of Western Canada but has been demonstrated to be rare in Ontario, where the 15-ADON chemotype dominates [18]. The presence of DON-3G increases the risk of human exposure to DON because the sugar is cleaved off by the gut microbiome in both humans and animals [19]. Other *Fusarium* species, such as *F. sporotrichioides* (T-2/HT-2 toxins) and *F. avenaceum* (beauvericin, enniatins), can also be common depending on weather conditions [20,21]. Periodic and systematic monitoring of mycotoxins in wheat and corn is essential to detect and track changes in contamination patterns and fungal populations. Climate change has emerged as a significant factor influencing mycotoxin distribution and prevalence. In North America, the impact of climate change on mycotoxin contamination has been identified as one of the primary threats leading to increased exposure to these chemical toxins [22,23].

In Canada, regular monitoring for mycotoxins is conducted for exported wheat, most of which comes from Western Canada [24,25,26,27,28]. Because all the corn and much of the wheat produced in Ontario stays in Canada, this level of monitoring is less frequent. In this study, we report the results of a comprehensive LC-MS/MS analysis of wheat and corn samples taken from across the cereal-producing areas of Ontario in each of the growing seasons of 2015, 2016, and 2017. Aside from DON and ZEN, we responded to a recommendation by the 83rd Joint Expert Committee on Food Additives and Contaminants (JECFA), who issued a call for survey data on sterigmatocystin (STE) and diacetoxyscirpenol (DAS) [29,30].

A comprehensive multi-mycotoxin screening method was employed for the simultaneous detection and quantification of 33 distinct mycotoxins in a total of 319 grain samples, comprising 159 wheat and 160 corn specimens. Additionally, ergosterol levels were measured as a biomarker to assess the overall fungal biomass and infection severity in the samples.

## 2. Results

Weather conditions where the wheat and corn were grown in the three growing seasons varied considerably. The 2015 growing season was somewhat cooler and much wetter than the 30-year average, where some areas in Southwestern Ontario received over 200% of normal rainfall amounts between 30 May and 30 June [31]. The 2016 growing season was 1–2.5° warmer and ~10% drier. The 2017 growing season was slightly cooler and somewhat wetter than the 30-year average [32,33,34].

### 2.1. Wheat

**Figure 1:**
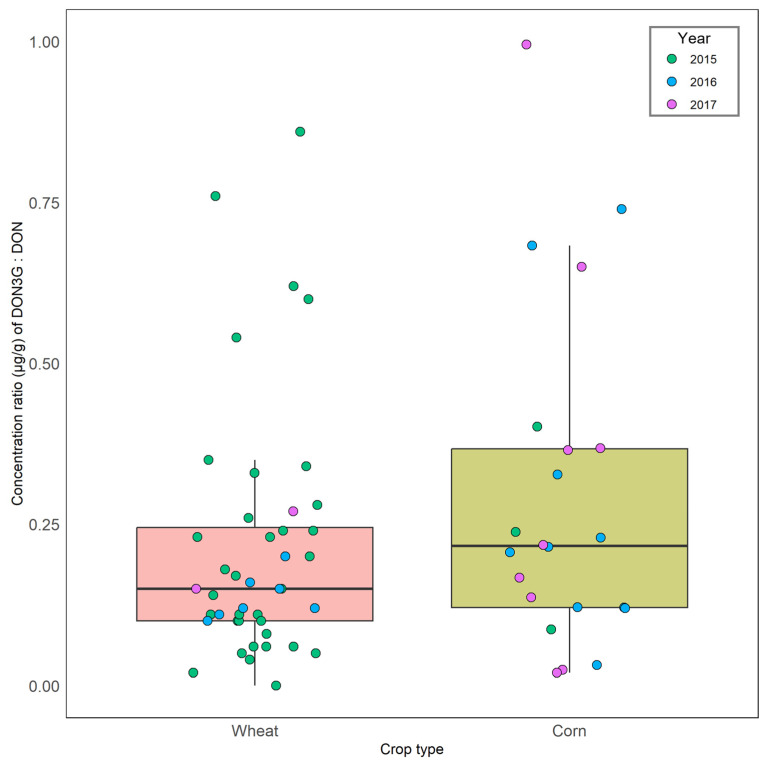
Concentration (µg/g) ratios of DON-3G:DON in wheat and corn over the 2015, 2016, and 2017 sampling years.

The pattern of toxins in the samples varied by crop year. In 2015, 59 samples were analyzed. Of these, 36% had >1 µg/g DON (Table 1). In these samples, the concentration (µg/g) ratio of DON-3G:DON varied considerably, with an arithmetic mean of 0.238 ± 0.215 (Figure 1). Enniatins B and B1 co-occurred at similar concentrations in the wheat samples. More than 1 µg/g enniatin B + B1(combined) was present in 20% of the samples, but most samples in 2015 had modest (66.0 µg/kg ± 2.7 enniatin B, B1) median concentrations of both. Approximately one-third of the samples where beauvericin was detected had modest (0.06–0.75 µg/g ± 0.17) median concentrations of beauvericin present. Zearalenone (ZEN) was detected in 76% of the 2015 wheat samples, and 66% of the detected samples had low median concentrations (0.02–1.64 µg/g ± 0.33), with one sample having >1 µg/g. Most samples had low concentrations (0.005–0.412 µg/g ± 0.040) of the Alternaria metabolites alternariol, tentoxin, alternariol, and alternariol monomethyl ether. Low concentrations (0.11–1.32 µg/g ± 0.22) of fusaric acid were also common.

**Figure 2:**
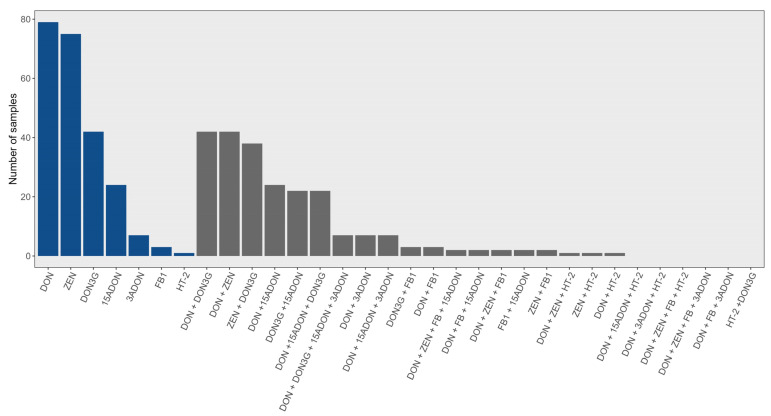
Number and distribution of mycotoxins (blue) and co-occurring mycotoxins (grey) in wheat samples between 2015 and 2017.

Four wheat samples were positive for STE in 2015, with a range of 0.14–9.42 µg/g, where two of the four hits were above 5 µg/g. In 2016 and 2017, 50 samples were analyzed. Sterigmatocystin was not detected in any of the 2016 and 2017 wheat samples. Diacetoxyscirpenol was not detected at all between 2015 and 2017. Considering all the toxins found in the wheat samples over three years, DON and ZEN were by far the most common and were almost always together (Figure 2). The precursor 15-ADON was also frequently detected (Table 1, Figure 2). The full dataset is found in the supplementary.xls file.

**Figure 3:**
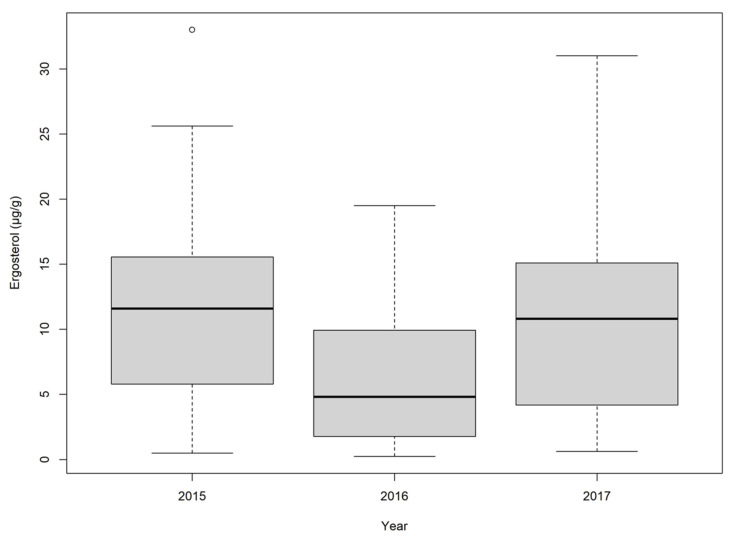
Ergosterol (µg/g) in wheat samples between 2015 and 2017. Outlier values (°) are shown as points outside of the maximum or minimum whisker of the dataset.

The median ergosterol values in wheat varied slightly between the three sampling years (Figure 3, Supplementary Table S2). The wheat samples from 2015 (10.7 µg/g) and 2017 (10.8 µg/g) were similar in both median values, positive hits (96% of samples in 2015, 100% in 2017), and maximum reported values (33.0 µg/g in 2015 and 31.0 µg/g in 2017). The wheat samples from 2016 had lower overall ergosterol by comparison, with a median concentration of 4.47 µg/g, and a maximum reported value of 19.5 µg/g. Though the concentrations of ergosterol were lower, the 2016 wheat samples were still comparable to 2015 and 2017 in the number of samples with quantifiable ergosterol (94% of samples). Pearson’s correlation of ergosterol showed a strong positive correlation (r = 0.95, *p* = 3.25 × 10^−3^) with STE, as well as a moderately positive relationship with DON (r = 0.44. *p* = 7.18 × 10^−5^).

### 2.2. Corn

In 2015, 44 samples were analyzed. Of these, DON was detected in 36% of the samples, where three samples had >1 µg/g DON (Table 2). Most of these had low (0.01–1.24 µg/g ± 0.33) ZEN except one sample at >1 µg/g. Beauvericin was detected in 18% of the 2015 corn samples. The majority of the samples with beauvericin had low concentrations (0.03–0.34 µg/g ± 0.13), except for one sample which had > 5 µg/g. The other toxins measured were largely absent.

In 2016, 51 samples were analyzed. Of these, 39% had detectible DON, where six samples were >1 µg/g, including two above 10 µg/g. The concentration (µg/g) ratio of DON-3G:DON was 0.282 ± 0.241 (Figure 1). Zearalenone was present in 60% of the samples, with one having > 1 µg/g. Of the 2016 samples, 16% of them had beauvericin, where detected samples had modest (0.03–6.43 µg/g ± 2.23) concentrations, with two samples at >2 µg/g.

In 2017, 65 samples were analyzed. Of these, 23% had DON, where six samples were >1 µg/g, including two samples that were >10 µg/g. The concentration (µg/kg) ratio of DON to DON-3G was 0.329 ± 0.322. Zearalenone was detected in 48% of the 2017 samples, with most samples having low concentrations of zearalenone (0.01–1.39 µg/g ± 0.31), with two samples having > 1 µg/g. Only four samples had detectible beauvericin, with two samples having > 1 µg/g.

Fumonisins were generally found in low concentrations overall (35.4 µg/kg–1.59 µg/g ± 0.261 µg/g for FB_1_, FB_2_ and FB_3_) between 2015 and 2017, and were uncommon across all three years of the study. The sole exception was a single sample from 2016, which contained 1.6 µg/g of FB_1_. Fusaric acid (FUSA) was uncommon in 2015 and had only a single detection (318 µg/kg). However, FUSA was detected in some samples in 2016 (0.107–1.180 µg/g ± 0.385) and in 2017 (0.199–2.060 µg/g ± 0.764). The full dataset is found in the Supplementary Materials.

**Figure 4:**
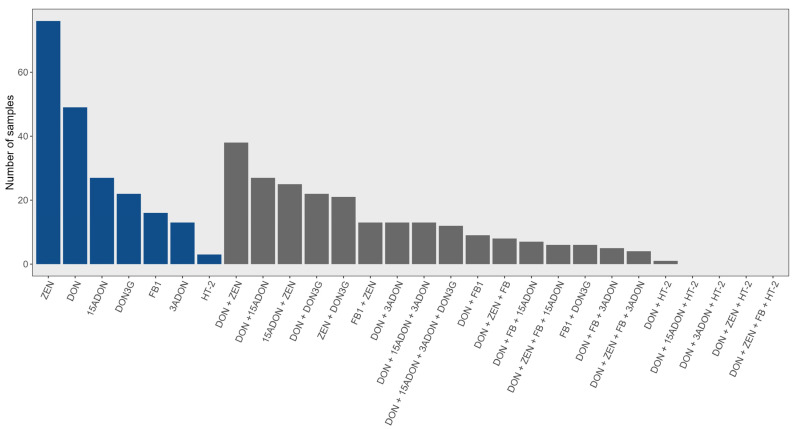
Number and distribution of mycotoxins (blue) and co-occurring mycotoxins (grey) in corn samples between 2015 and 2017.

Considering all the toxins that were detected in the corn, DON, 15-ADON, and ZEN were by far the most common (Figure 4). As noted, fumonisin occurred sporadically, mostly in low µg/kg concentrations (Table 2).

A number of compounds were not detected, such as diacetoxyscirpenol, primarily from *F. sambucinum* [35], and the *Penicillium* toxins griseofulvum and mycophenolic acid. Other *Penicillium* compounds, including penitrem A and viridicatin as well as the *Aspergillus versicolor* toxin sterigmatocystin, were infrequently detected in low (µg/kg) abundance (Table 2, Supplementary Tables S1 and S2).

**Figure 5:**
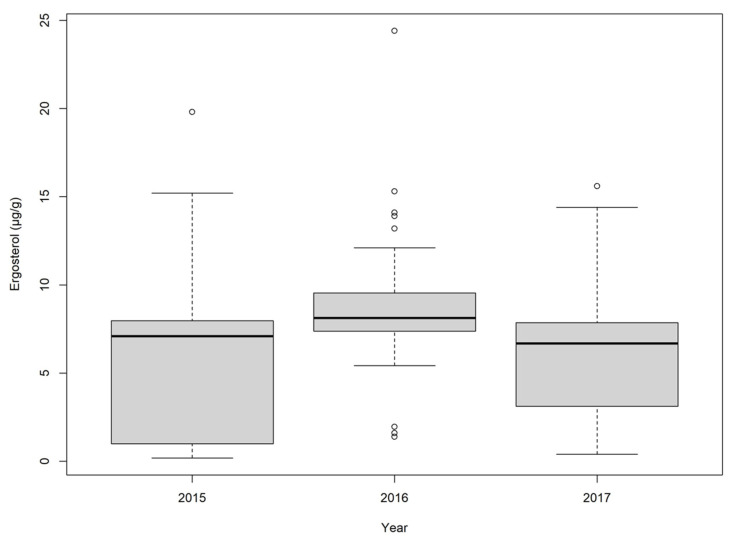
Ergosterol (µg/g) in corn samples between 2015 and 2017. Outlier values (°) are shown as points outside of the maximum or minimum whisker of the dataset.

Ergosterol in the corn samples was typically lower than in the wheat samples, where fewer samples had detectable levels, especially in 2017 (Figure 5, Supplementary Table S2). The samples from 2015 and 2016 had comparable detections (88.6% of samples in 2015, 82.3% in 2016) and median values (6.00 µg/g in 2015, 7.85 µg/g in 2016). By contrast, in 2017, only 55.3% of the corn samples had detectable levels of ergosterol, with a median value of 1.24 µg/g. Pearson’s correlation showed only a moderate correlation of ergosterol value to DON (r = 0.59, *p* = 4.41 × 10^−5^), D3G (r = 0.57, *p* = 9.39 × 10^−3^), and ZEN (r = 0.42, *p* = 8.98 × 10^−4^).

## 3. Discussion

Wheat becomes susceptible to head blight when warm and wet conditions coincide with anthesis, and corn becomes susceptible to ear rot if those conditions occur around silk emergence. Corn is susceptible to Fusarium kernel rot and the accumulation of fumonisins under hot dry conditions, often combined with insect stress [36]. The impacts of temperature and rainfall are clearly seen in the data from the two crops over the three years of the survey. 

As noted, DON remains the dominant mycotoxin in Ontario-grown wheat, and this has not changed since the mid-1980s, nor since the severe epidemics of Gibberella ear rot from the mid-1990s [37]. Generally, modest concentrations of the cyclic peptides, enniatins, and beauvericin were seen in both crops. This is consistent with other recent studies in Ontario of both wheat and corn [38,39]. Strains of *F. verticillioides* from Ontario were shown to produce fumonisins shortly after their discovery in the early 1990s [40]. Despite historical concerns, fumonisin contamination remains relatively uncommon. Nevertheless, the risk persists, particularly during warm summers, necessitating continued vigilance. A recent study of mycotoxins in corn harvested in Michigan indicated a similar pattern, except fumonisin was more common and in higher average concentrations [41]. As noted, the minor compounds from *Penicillium* species and STE were uncommon in both crops over the three years. This is similarly consistent with the recent study of wheat in Ontario and in samples of grain corn in the USA [39,42]. This, along with fact that DAS was not observed in any samples, helps to address the question of what the risk is of these potentially emerging mycotoxins to grains in Canada. It is clear that there is currently very little to no risk of STE or DAS contamination during the years studied for this survey. This finding further reiterates the importance of DON in Canada and focuses the need for devoting our attention and resources to the continued monitoring and management of DON.

Tothill et al. (1992) noted significant correlations between ergosterol values at higher water activity (a_w_) values (0.95) [43]. Similarly, Abramson et al. (2005) noted increased ergosterol after the wheat moisture content (mc) increased from 3.9–8.4 µg/g at 16% mc to 3.9–55.5 µg/g at 20% mc in stored Manitoba wheat samples [44]. The samples from 2015 in the current study had the highest recorded rainfall between the sampling years and also the maximum reported ergosterol values. The ergosterol ranges for wheat (0.24–33 µg/g) and corn (0.19–24 µg/g) found in this study align with the ranges reported by Tittlemier et al. (2020) for Canadian oat sample shipments taken between 2014 and 2017 (1.77–37 µg/g) [45]. Perkowski et al. (2008) reported lower ergosterol values (0.4–3.40 µg/g) in Polish wheat samples [46]. In contrast, Pietri et al. (2004) reported higher ergosterol values (0.3–174.8 µg/g) from a multi-year study of Italian corn samples, with significant correlations shown between ergosterol and high concentrations of DON (*p* < 0.01), ZEN (*p* < 0.01) and FB_1_ (*p* < 0.001) [47] 

The largest difference over the three years of this study was the extremely variable DON-3G concentrations found. In some cases, DON-3G was >50% of the DON concentration in wheat in the 2015 samples, where DON was >1 ppm (Figure 1). At the time of the original report of the existence of a DON glycoside in wheat in 1986 [48], DON-3G was present in low concentrations or not detectable in Ontario wheat and corn. Based on the available data, wheat cultivars have higher ratios of DON-3G to DON in Europe than in the USA [49]. Wheat cultivars from Europe also tend to have higher relative amounts of DON-3G [50]. In both humans and swine, the gut microbiome can cleave the sugar off, thus increasing their exposure to DON [51,52]. Because of this, the European regulation for DON is a value comprising the sum of DON, DON-3G, and the two acetates 15- and 3-ADON [53]. The ratios of DON-3G to DON in the positive corn samples were comparable to those reported by Blackwell et al. (2022) [54].

Our initial interest in the samples was to monitor for the genetic changes of the *F. graminearum* population in Ontario cereals [18,37]. The present work attempts to determine if any major changes in the mycotoxins profile have occurred by comparing the present findings to studies published over the past 40 years. During this study period, the measured temperature change in Southern Ontario has increased by >1 °C [15]. The present data suggest little change to the overall mycotoxin profile in cereals so far. However, the data will permit this question to be asked again in a decade or so from now. In Europe, growing concern has emerged regarding the potential health risks associated with the co-occurrence of low-level mycotoxins and minor secondary metabolites in food and animal feed [55]. Much of this is based on in vitro tests of limited applicability to human and animal health [56]. The one example of interaction that has human health relevance is the combination of aflatoxin and fumonisin. The impact of co-exposure of a genotoxic carcinogen with a powerful cancer promoter has been clearly demonstrated in relevant animal models, and the mechanism is clear [57]. Mixtures of the major metabolites from *F. graminearum* that co-occur with DON (15-ADON, culmorin, sambucinol, and dihydroxycalonectrin) in typical concentrations do not result in an increased effect over DON in swine [58]. The ZEN and DON quantified in the current study commonly co-occurred in corn, albeit at levels that were below the guidelines for swine. Early studies demonstrated that the effect of ZEN on swine was antagonized by co-exposure [59,60]. This reflects the well-understood feed refusal effect of DON on swine.

## 4. Conclusions

The analysis of the wheat and corn samples from 2015 to 2017 revealed that DON, ZEN, and 15-ADON were the most commonly detected mycotoxins from farms in Ontario, Canada. This trend remains unchanged for the region when comparing the present findings to data published over the last 40 years. However, what has changed is the occurrence of DON-3G, which was uncommon in the past. The implications of this change in terms of human exposure need further study, preferably by a urinary biomarker study. In addition, the analysis revealed that there was little risk of STE and DAS contamination in maize and small grains over the sampling period and fulfilled the survey recommendation proposed by JECFA. However, there needs to be continued surveillance of STE, DAS, and *Fusarium* trichothecenes in small grains and maize as regional trends may shift as average temperatures and rainfall amounts increase. 

## 5. Materials and Methods

### 5.1. Corn and Wheat Sample Collection 

Samples were collected as described in Crippin et al. (2020) [37]. Briefly, 160 corn and 159 wheat samples (90–100 g) were collected in 2015, 2016, and 2017 from the southern, central, and eastern regions of Ontario, Canada. The samples were collected by the Ontario Corn Committee and Kent Corn Committee from experimental plots at the University of Guelph’s Ridgetown Campus. Grain samples were also collected from annual Fusarium head blight and Gibberella ear rot surveys that were conducted by the Ontario Ministry of Food and Agriculture and the University of Guelph, Ridgetown Campus. It is important to note that in Ontario there is a rotation of soybean, corn, and wheat crops, with cereals following soybeans. The samples were all stored under air drying conditions at −20 °C at the University of Guelph, Ridgetown Campus, until being sent to Agriculture Agri-Food Canada in London.

All the samples were air dried and stored at −20 °C until being processed in 2023. The corn and wheat samples were ground using the Retsch Twister Mill with a 1 mm sieve. The ground subsamples were stored in paper envelopes at −20 °C until extraction.

### 5.2. Mycotoxin Extraction

The ground corn and wheat samples (200 mg ± 20 mg) were extracted with 1 mL 79/20/1 acetonitrile/H_2_O/acetic acid [61,62,63,64,65]. The samples were sonicated for 15 min and then shaken at 1400 rpm on an Eppendorf Thermomixer C (2.0 mL sample block) at 30 °C for 20 min, in accordance with Kelman, 2022, and Teeter-Wood, 2024 [66,67]. The samples were first incubated at 4 °C for 10 min before being centrifuged at 4 °C at 8000 rpm for 8 min. The supernatant was diluted 1:1 with water and filtered through 0.45 µM PTFE syringe filters into 250 µL polypropylene snap cap vials (Agilent Technologies, Santa Clara, CA, USA) for an LC-MS analysis [64].

### 5.3. LC-MS/MS Analysis and Quantification of Mycotoxins

The corn and grain samples were initially pre-screened by an LC-MS analysis on an Agilent 1290 HPLC that was coupled to a Q-Exactive Quadrupole Orbitrap Mass Spectrometer, as previously described [66]. All the grain samples were screened for NX and 3ANX toxins, but these were not detected and, thus, not included in the quantification. A mycotoxin quantification by LC-MS was performed on a Thermo Fisher Vanquish HPLC that was coupled to a Thermo Fisher TSQ Altis triple quadrupole mass spectrometer (Waltham, MA, USA). Separation was achieved using a Zorbax Eclipse Plus RRHD C18 column (2.1 × 100 mm, 1.8 µM, Agilent Technologies) maintained at 35 °C. The sample extracts were analyzed using water with 0.1% formic acid (mobile phase A) and acetonitrile with 0.1% formic acid (mobile phase B) (Optima grade, Fisher Scientific, Lawn, NJ, USA). Briefly, B was held at 2% for 0.75 min and increased to 22% B over 0.5 min. Then, B was slowly increased to 35% over 2.75 min before ramping to 100%. Finally, B was held at 100% for 2.5 min before returning to 2% over 0.5 min. Each sample was injected with 5 µL using a flow rate of 0.3 mL/min. The samples were analyzed using a HESI in SRM mode using the following spray conditions: ion spray voltage, 3500 V; sheath gas, 35 units; auxiliary gas, 10 units; sweep gas, 1 unit; transfer tube temperature, 325 °C; and vaporizer temperature, 350 °C. The calibration standards were prepared neat in 50/50 acetonitrile/water over low (0.2–20 ng/mL) and high (50–800 ng/mL) concentration ranges. The instrumental limits of detection and quantification were determined using the standard deviation of y-intercepts of regression lines following ICH guidelines [68]. The methods were validated in corn and wheat sample matrices. The clean, blank corn and wheat material required for SSE (signal suppression and enhancement) and recovery (R_E_) experiments were determined by extraction and an LC-MS analysis of the provided samples (as previously described) and by selecting the samples with the lowest detected amounts of mycotoxins in the screening method (Supplementary Table S1). The extraction recovery experiments were conducted at 500 µg/kg into blank matrix material (corn, wheat) using 200 ± 20 mg of clean corn and wheat sample matrices. The spiked samples were vortexed for 60 s before being dried for 30 min at room temperature. The control and spiked samples were extracted as previously described [61,63,64,65]. After centrifugation, the supernatant was removed and the control sample supernatants were similarly spiked with 500 µg/kg of the mycotoxin mixture. The supernatant (400 µL) was removed and diluted 1:1 with water and filtered with 0.45 µM PTFE syringe filters into 250 µL polypropylene snap cap HPLC vials for an LC-MS analysis. All the peak areas were integrated for the quantifier ion listed in Supplementary Table S1. Labeled standards of U[^13^C_18_] Sterigmatocystin (25.1 µg/mL, Romer Labs); U[^13^C_34_] Fumonisin B_1_ (25 µg/mL, Romer Labs); U[^13^C_19_] Diacetoxyscirpenol (25.1 µg/mL, Romer Labs); U[^13^C_15_] Deoxynivalenol (25 µg/mL, Romer Labs); U[^13^C_20_] Ochratoxin A (10 µg/mL, Romer Labs); and U[^13^C_18_] Zearalenone (25.1 µg/mL, Romer Labs) were used as quality controls for quantifications. Briefly, the internal standard (IS) spiking solution was prepared as a dilution from stock solutions in 50/50 acetonitrile/H_2_O. The wheat and corn samples (200 mg ± 0.2) were spiked with 5 µL of IS to final concentrations of 10 µg/kg OTA and STE; 100 µg/kg FB_1_, ZEN, and DAS; and 500 µg/kg DON on grain. After spiking, the corn and wheat samples were air-dried under a gentle stream of nitrogen for 15 min prior to extraction (5.2). All samples with positive hits for OTA, STE, FB1, ZEN, and DON were validated using their internal standard prior to quantification by external calibration by their unlabeled standard.

### 5.4. Ergosterol Extraction and Quantification by LC-MS/MS

The LC-MS analysis of ergosterol was performed using an APCI source, as previously described [66], with a minor modification to the chromatography to adjust for the d7-cholesterol internal standard. The corn and wheat samples were weighed (200 mg ± 20 mg) into anaerobic tubes and spiked at 50 µg/g d7-cholesterol (Sigma Aldrich, prepared in 1 mg/mL stocks in acetone) and allowed to dry for 15 min before extraction. The extraction solution was prepared per Tittlemier et al., 2020, and Dong et al., 2006, using 10% potassium hydroxide in methanol [45,69]. Two mL of the 10% KOH was added to the samples that were spiked with an internal standard, which were then vortexed briefly for 15 s, capped, and saponified at 90 °C in a water bath for 90 min. Following solution neutralization with hydrochloric acid, samples were extracted 1:1 (*v*:*v*) with hexane. The organic portions were removed and evaporated to dryness and then reconstituted in 1 mL optima grade acetonitrile. Samples were diluted 100× with acetonitrile and syringe filtered with 0.45 µm PTFE into HPLC vials for an LCMS analysis. Separation was achieved using a Zorbax Eclipse Plus C8 column (2.1 × 50 mm × 1.8 µm; Agilent Technologies) using mobile phase A (Optima grade water + 0.1% formic acid) and mobile phase B (Optima grade acetonitrile + 0.1% formic acid). B was held at 30% for 30 secs, increased to 100% over 3 min, and held at 100% B for 3.5 min, returned to 30% B over 1.5 min, and held for 1 min before the next injection. Samples were quantified using matrix-matched calibration curves in clean wheat and corn matrices up to 700 µg/g with a reported extraction efficiency of 92%. Ergosterol LOD and LOQ (10 ng/mL) were determined from 5 repeated injections with % RSD < 25% that were run on the Q-Exactive Orbitrap with an APCI source, with an MQL of 0.054 mg/kg. Pearson’s correlation (α = 0.05) was performed in R using the Hmisc [70] package to assess whether ergosterol was correlated to mycotoxin concentration (µg/g).

## Figures and Tables

**Table 1:** Quantification ^1^ of mycotoxins and secondary metabolites in 159 wheat samples from Ontario farms from 2015–2017.

	2015, *n* = 59		2016, *n* = 50		2017, *n* = 50	
	Median (# ^2^ Hits) µg/kg	Range (Min–Max) µg/kg	Median (# ^2^ Hits) µg/kg	Range (Min–Max) µg/kg	Median (# ^2^ Hits) µg/kg	Range (Min–Max) µg/kg
Deoxynivalenol (DON)	948 (53)	20.1–14,600	537 (16)	8.96–8070	109 (11)	18.2–563
15-Acetyldeoxynivalenol (15-ADON)	82.3 (45)	38.7–165	77.0 (4)	70.0–165		
3-Acetyldeoxynivalenol (3-ADON)	113 (6)	81.6–148	118 (1)			
Deoxynivalenol-3-glucoside (DON-3G)	271 (33)	35.5–67	315 (7)	114–993	107 (2)	85.1–128
Zearalenone (ZEN)	28.4 (45)	16.5–1640	19.3 (13)	16.2–59.5	18.5 (17)	16.1–4670
HT-2 toxin (HT-2)	237 (1)					
Fumonisin FB_1_ (FB_1_)	42.8 (3)	41.8–47.1				
Fumonisin FB_2_ (FB_2_)	33.6 (3)	33.6–34.2	34.0 (1)			
Fumonisin FB_3_ (FB_3_)	46.3 (3)	45.9–47.4	48.3 (1)			
Sterigmatocystin (STE)	3630 (4)	135–9420			1420 (2)	322–2510
α-cyclopiazonic acid (αCPA)	121 (7)	118–163	118 (1)		117 (2)	117–117
Mycophenolic acid (MPA)	494 (2)	132–855				
Penitrem A (PENTA)	51.3 (3)	51.0–53.9			51.6 (1)	
Roquefortine C (ROC)	26.2 (5)	25.1–42.4			28.2 (2)	26.9–29.4
Viridicatin (VIRI)	54.9 (4)	52.3–63.7			60.6 (2)	58.2–63.0
Altenuene (ALT)	13.6 (12)	11.6–18.6	14.2 (3)	12.9–14.4	11.9 (1)	
Tentoxin (TEN)	41.9 (40)	4.73–120	45.6 (9)	2.70–110	16.7 (11)	3.05–96.5
Alternariol (AOH)	23.5 (42)	15.7–412	23.2 (12)	17.2–85.7	19.3 (8)	18.3–36.1
Alternariol monomethyl ether (AME)	20.4 (42)	16.0–84.2	20.6 (12)	17.5–39.2	19.8 (8)	17.5–27.0
Beauvericin (BEA)	118 (21)	9.27–748	324 (5)	52.4–448	80.4 (4)	50.0–554
Enniatin B (ENNB)	65.6 (59)	5.29–6500	8.84 (41)	5.30–1580	6.59 (38)	3.08–50.4
Enniatin B1 (ENNB1)	66.8 (59)	22.6–4100	26.2 (41)	22.5–677	23.0 (39)	22.0–50.0
Fusaric acid (FUSA)	116 (34)	112–230	115 (12)	112–133	114 (12)	111–216

^1^ Only samples above the limit of quantification (LOQ) are included. Instrumental LOQ values are reported in the Supplementary Table S1. ^2^ Number (#) of hits or detections > LOQ.

**Table 2:** Quantified ^1^ mycotoxins and secondary metabolites in 160 corn samples from Ontario farms between 2015 and 2017.

	2015, *n* = 44		2016, *n* = 51		2017, *n* = 65	
	Median (# ^2^ Hits) µg/kg	Range (Min–Max) µg/kg	Median (#^2^ Hits) µg/kg	Range (Min–Max) µg/kg	Median (# ^2^ Hits) µg/kg	Range (Min–Max) µg/kg
Deoxynivalenol (DON)	125 (16)	15.5–3760	393 (20)	17.9–17,600	319 (15)	63.8–18,500
15-Acetyldeoxynivalenol (15-ADON)	126 (4)	63.9–178	195 (13)	62.2–335	128 (10)	57.3–1080
3-Acetyldeoxynivalenol (3-ADON)	136 (1)		302 (6)	92.7–1184	502 (5)	218–981
Deoxynivalenol-3-glucoside (DON-3G)	331 (4)	107–1300	447 (9)	75.6–6650	176 (9)	89.0–4930
Zearalenone (ZEN)	16.5 (20)	12.2–1790	13.1 (30)	12.0–1620	14.1 (31)	12.0–1390
HT-2 Toxin (HT-2)	1260 (2)	901–1630	1240 (1)		883 (1)	
Fumonisin FB_1_ (FB_1_)	47.4 (4)	46.0–52.1	51.7 (10)	45.2–1600	55.0 (4)	45.4–76.6
Fumonisin FB_2_ (FB_2_)	35.8 (4)	35.6–38.2	36.8 (7)	35.5–118	36.3 (2)	35.4–37.3
Fumonisin FB_3_ (FB_3_)	54.0 (2)	53.4–54.7	55.6 (7)	53.3–101	55.0 (1)	
Sterigmatocystin (STE)	750 (1)					
α-cyclopiazonic acid (αCPA)			100.0 (1)			
Penitrem A (PENTA)	54.4 (1)					
Roquefortine C (ROC)	79.7 (6)	36.8–371			28.3 (1)	
Viridicatin (VIRI)	139 (3)	71.4–250				
Altenuene (ALT)	20.1 (8)	13.8–28.3	17.5 (4)	12.1–41.1	17.1 (3)	12.5–26.3
Tentoxin (TEN)			4.12 (2)	3.81–4.42		
Alternariol (AOH)	69.4 (16)	24.1–214	27.4 (19)	21.3–218	28.0 (16)	19.0–177
Alternariol monomethyl ether (AME)	16.1 (2)	14.6–17.6	74.5 (3)	23.3–332		
Beauvericin (BEA)	168 (8)	34.5–5670	180 (8)	34.0–6430	605 (5)	77.0–13,000
Enniatin B (ENNB)	5.27 (26)	4.81–6.78	5.03 (10)	4.83–5.54	5.33 (24)	4.81–8.40
Enniatin B1 (ENNB1)	32.8 (1)		25.3 (1)		35.1 (1)	
Fusaric acid (FUSA)	318 (1)		411 (8)	107–1180	982 (6)	199–2060

^1^ Only samples above the limit of quantification (LOQ) are included. Instrumental LOQ values are reported in the Supplementary Table S1. ^2^ Number (#) of hits or detections > LOQ.

## Data Availability

Raw data are available on request from the authors.

## Supplementary Materials

The following supporting information can be downloaded at https://www.mdpi.com/article/10.3390/toxins16080372/s1. Supplementary.pdf: Table S1: Instrumental LODs and LOQs and associated quantifier/qualifier ions of analyzed mycotoxins; Table S2: MRM transition information for ^13^C labeled mycotoxin standards; Table S3: Ergosterol concentration summary (µg/g) in wheat and corn between 2015 and 2017; Figure S1: Pearson correlation coefficients (r) for 159 wheat samples, (α = 0.05); Figure S2: Pearson correlation coefficients (r) for 160 corn samples, (α = 0.05); Figure S3: DON:DON3G concentration ratio in corn samples by year (2015–2017); Figure S4: Pearson correlation coefficients (r) for 160 corn samples, (α = 0.05). Wheat and corn data.xlsx: 2015–2017 wheat data, 2015–2017 corn data, mycotoxin abbreviations.
