# Supplementary material for: A Multi-Year Study of Mycotoxin Co-Occurrence in Wheat and Corn Grown in Ontario, Canada
Source: Toxins (Basel). 2024 Aug 22;16(8):372. doi: 10.3390/toxins16080372 (PMC11359917; doi:10.3390/toxins16080372)
Supplement: Supplementary file 1 [file toxins-16-00372-s001.zip › Supplementary_Aug12.pdf]

Table S1- Instrumental LODs and LOQs and associated quantifier/qualifier ions of analyzed mycotoxins

Analyte	RT (mins)	Ion type	Precursor *m/z*	Rf (V)	Quan/Qual	Collision energy (V)	R_E (%)	SSE_{corn} (%)	SSE_{wheat} (%)	LOD[a] (ng/mL)	LOQ[a] (ng/mL)
Deoxynivalenol-3-glucoside (DON-3G)	2.26	$[M+FA]^-$	503.17	91.3	427.13/457.16	19/14	52	85	90	1.84	6.08
NX toxin (NX)	2.41	$[M+H-H_2O]^+$	265.14	41.3	247.08/159.80	11/20	86	96	94	3.62	11.9
Deoxynivalenol (DON)	2.48	$[M+H]^+$	297.11	37.8	249.01/231.01	10/11	91	73	92	0.971	3.20
Fusaric acid (FUSA)	3.09	$[M+H]^+$	180.10	40.4	134.01/162.01	16/11	47	97	89	1.63	5.37
15-Acetyldeoxynivalenol (15-ADON)	3.59	$[M+H]^+$	339.14	59.1	261.08/321.17	12/8	82	86	98	1.89	6.24
3-Acetyldeoxynivalenol (3-ADON)	3.76	$[M+H]^+$	339.14	53.2	261.08/231.08	12/11	83	79	108	0.971	3.21
Fumonisin B_1 (FB_1)	4.63	$[M+H]^+$	722.39	127.1	334.01/352.01	41/36	71	111	120	0.630	2.08
Ergocryptine (ERGCRY)	4.69	$[M+H]^+$	576.31	75.4	558.33/268.16	15/24	77	115	117	0.546	1.80
Altenuene (ALT)	4.70	$[M+H]^+$	293.10	39	257.08/239.01	13/20	81	138	149	0.161	0.531
Ochratoxin A (OTA)	4.76	$[M+H]^+$	404.08	64.0	238.90/358.00	23/13	72	122	86	0.799	2.63
Roquefortine C (ROC)	4.80	$[M+H]^+$	390.19	78.9	193.08/322.08	29/20	81	128	139	0.556	1.84
Ergocristine (ERGCRI)	4.83	$[M+H]^+$	610.30	76	592.33/268.16	14/25	80	81	122	0.553	1.83
Ergocryptinine (ERGCRYN)	4.89	$[M+H]^+$	576.31	75.4	558.33/268.16	15/24	77	114	111	0.564	1.86
Ergocristinine (ERGCRIN)	5.03	$[M+H]^+$	610.30	76	592.33/268.16	14/25	80	93	133	0.548	1.81
Fumonisin B_3 (FB_3)	5.03	$[M+H]^+$	706.40	127.7	336.33/318.33	36/37	64	89	103	0.627	2.07
Diacetoxyscirpenol (DAS)	5.18	$[M+H]^+$	367.20	57	349.10/307.10	8/10	88	105	84	0.153	0.506
Fumonisin B_2 (FB_2)	5.19	$[M+H]^+$	706.40	130.1	336.33/318.33	36/38	75	131	138	0.596	1.97
HT-2 toxin (HT-2)	5.50	[M+Na]+	447.19	80	345.00/285.00	18/20	84	27	98	1.89	6.27
Alternariol (AOH)	5.52	$[M-H]^-$	257.04	147.7	213.00/147.00	23/33	66	59	66	0.215	0.708
Tentoxin (TEN)	5.54	$[M-H]^-$	413.21	108.6	271.00/141.00	17/21	89	114	111	0.145	0.479
o-methyl-sterigmatocystin (omeSTE)	5.92	$[M+H]^+$	339.08	111.4	306.08/324.08	28/23	88	82	105	0.679	2.24
Mycophenolic acid (MPA)	5.93	$[M+H]^+$	321.13	52.8	207.08/303.08	21/10	61	108	85	0.708	2.34
Griseofulvin (GRIS)	5.94	$[M+H]^+$	353.20	125.6	285.08/215.01	17/20	91	131	121	0.161	0.532
Viridicatin (VIRI)	5.98	$[M+H]^+$	238.08	91.2	165.08/192.08	36/25	60	86	121	0.150	0.495
Citrinin (CIT)	6.02	$[M+H]^+$	251.09	53	191.08/203.00	26/34	39	123	103	1.64	5.40
T-2 toxin (T-2)	6.25	$[M+Na]^+$	489.21	128	245.00/387.00	28/22	68	61	38	12.9	42.6
Alternariol monomethyl ether (AME)	6.46	$[M-H]^-$	271.06	113.6	228.00/227.00	30/37	79	78	63	0.157	0.519
Zearalenone (ZEN)	6.49	$[M-H]^-$	317.13	113.6	131.00/273.00	29/19	95	89	63	0.254	0.839
Sterigmatocystin (STE)	6.68	$[M+H]^+$	325.06	86.5	281.08/310.00	37/25	88	92	85	0.155	0.511
α-cyclopiazonic acid (αCPA)	6.98	$[M+H]^+$	337.15	67.6	196.08/182.08	22/18	77	72	61	0.671	2.21
Penitrem A (PENTA)	7.56	$[M+H]^+$	634.29	73.2	558.33/616.33	18/11	87	100	105	0.125	0.412
Beauvericin (BEA)	8.25	$[M+NH_4]^+$	801.74	75.8	244.16/134.08	31/54	86	133	90	2.39	7.87
Enniatin B (ENNB)	8.40	$[M+H]^+$	640.60	175	196.16/214.16	24/25	82	127	116	0.169	0.558
Enniatin B1 (ENNB1)	8.85	$[M+H]^+$	654.43	183	196.16/210.16	25/23	82	115	109	0.080	0.263

RT: Retention time; Rf: Radiofrequency; R_E: Recovery; SSE: Signal Suppression Enhancement; LOD: Limit of Detection; LOQ: Limit of Quantification

[a] LOD and LOQ calculated using standard deviation of y-intercepts of regression line following ICH guidelines (Borman, P. & Elder, D., 2017)[67]

Table S2- MRM transition information for ^{13}C labelled mycotoxin standards

^{13}C labelled mycotoxin	RT (mins)	Ion type	Precursor m/z	Rf (V)	Quan/Qual	Collision energy (V)
U[$^{13}C_{15}$] Deoxynivalenol	2.48	$[M+H]^+$	312.13	37.8	263.10	10
U[$^{13}C_{34}$] Fumonisin B_1	4.63	$[M+H]^+$	756.51	127.1	356.37/397.37	41/36
U[$^{13}C_{20}$] Ochratoxin A	4.76	$[M+H]^+$	424.15	64.0	250.2	23
U[$^{13}C_{19}$] Diacetoxyscirpenol	5.18	$[M+H]^+$	386.18	57.0	368.10/325.10	8/10
U[$^{13}C_{18}$] Zearalenone	6.49	$[M-H]^-$	335.14	113.6	140	29
U[$^{13}C_{18}$] Sterigmatocystin	6.68	$[M+H]^+$	343.07	86.5	297.0	37

Table S3- Ergosterol concentration summary (µg/g) in wheat and corn between 2015-2017

	Wheat			Corn		
µg/g	**2015**	**2016**	**2017**	**2015**	**2016**	**2017**
Median (n detected), µg/g	10.7 (57)	4.47 (47)	10.8 (50)	6.00 (39)	7.85 (42)	1.24 (36)
Range (min-max), µg/g	0.49 – 33.0	0.24 – 19.5	0.63 – 31.0	0.19 – 19.8	1.96 – 24.4	0.40 – 15.6

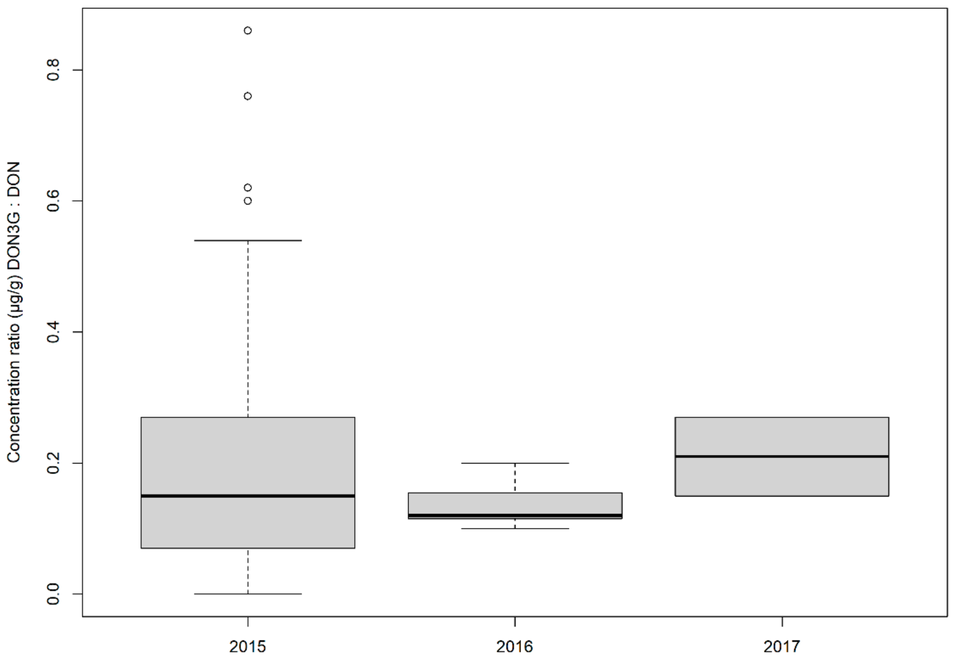

Figure S1- DON:DON3G concentration ratio in wheat samples by year (2015-2017)

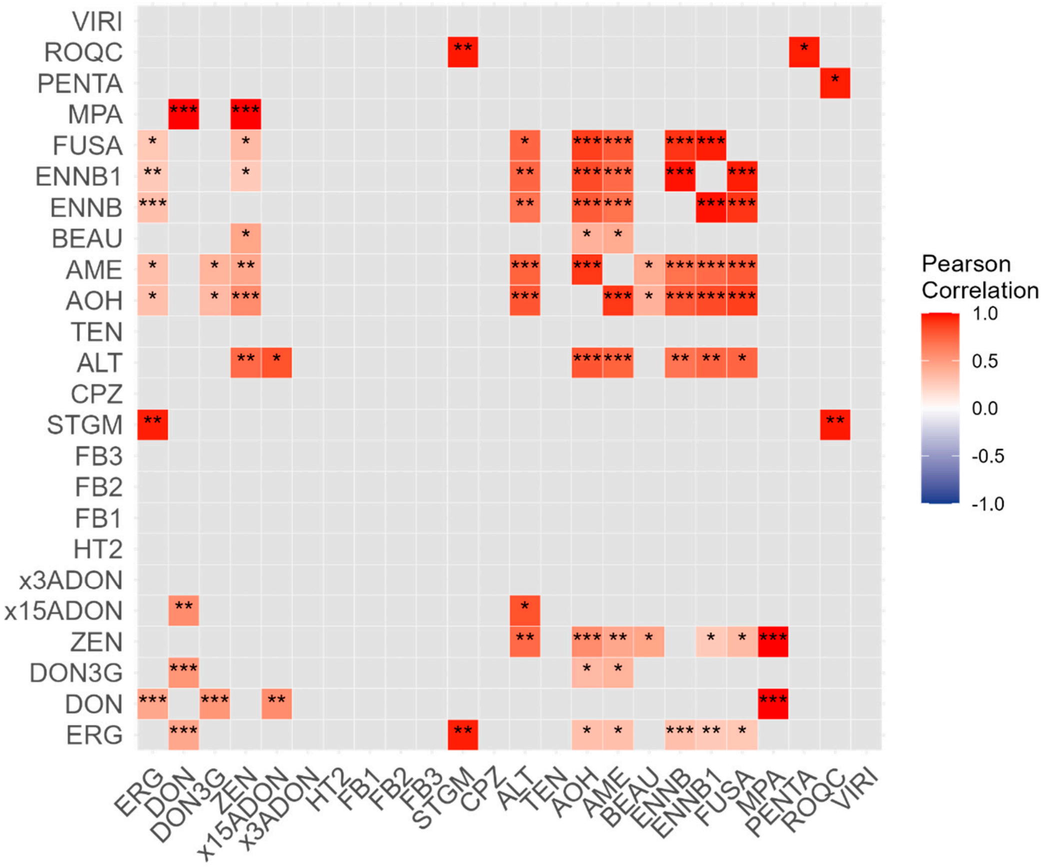

Figure S2- Pearson correlation coefficients (r) for 159 wheat samples, ($\alpha = 0.05$). * values indicate significance level (* [$p = 0.05$], ** [$p = 0.01$], *** [$p = 0.001$])

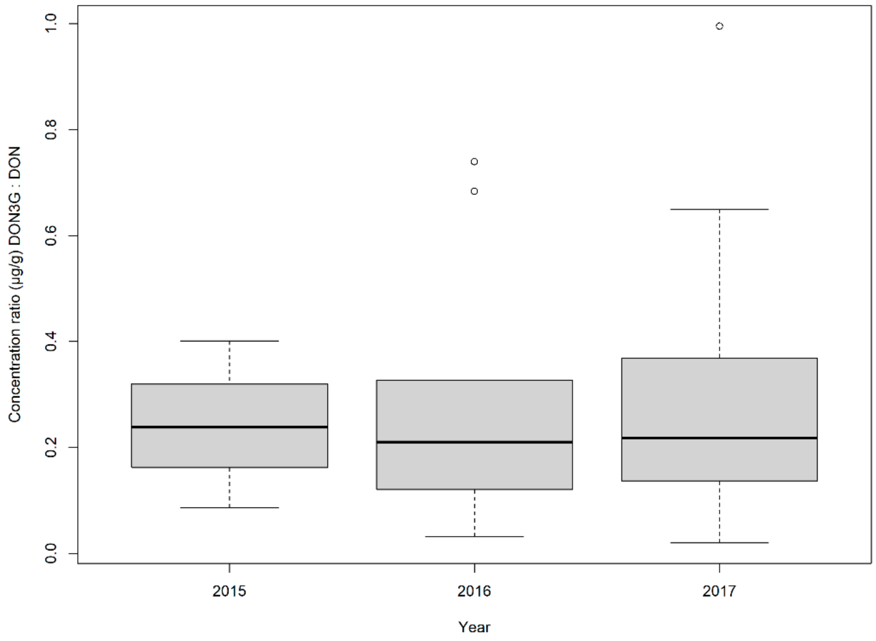

Figure S3- DON:DON3G concentration ratio in corn samples by year (2015-2017)

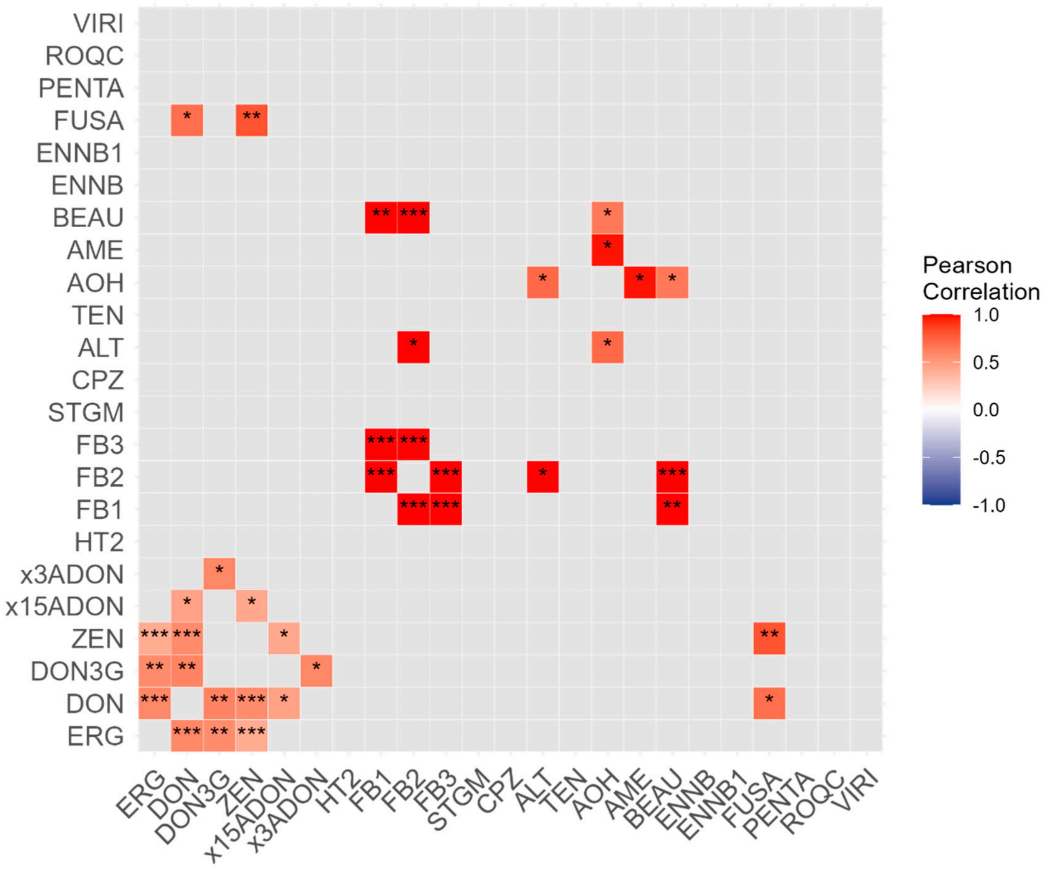

Figure S4- Pearson correlation coefficients (r) for 160 corn samples, ($\alpha = 0.05$). * values indicate significance level (* [$p = 0.05$], ** [$p = 0.01$], *** [$p = 0.001$])
